# 2404. Widespread emergence of infective endocarditis due to *Serratia* spp.: Results of a large multicenter cohort

**DOI:** 10.1093/ofid/ofad500.2024

**Published:** 2023-11-27

**Authors:** L Madeline McCrary, Sunish Shah, Douglas Slain, Bobbi Jo Stoner, Ashley H Marx, Asher J Schranz

**Affiliations:** Washington University in St. Louis, Chapel Hill, North Carolina; Antibiotic Management Program, UPMC Presbyterian Hospital, Pittsburgh, PA, Pittsburgh, Pennsylvania; West Virginia University, Morgantown, West Virginia; University of Kentucky, Lexington, Kentucky; University of North Carolina Medical Center, Chapel Hill, North Carolina; University of North Carolina, Chapel Hill, NC

## Abstract

**Background:**

Infective endocarditis (IE) due to *Serratia* spp. (S-IE) has historically been considered a rare entity and limited to case series. Typically linked to injection drug use (IDU), S-IE appears to be growing amidst the opioid crisis. Guidance on therapy of non-HACEK gram negative IE remains limited. Our goal was to assess trends in S-IE and evaluate management strategies.

**Methods:**

We retrospectively assessed adults treated for S-IE at 4 academic health systems in 4 US states from 2015-2021. Each site utilized different methods of identifying S-IE cases. Chart review was performed to assess management of cases. Multivariable logistic regression analyzed the association between antibiotic regimens and/or procedural management (defined as valve surgery or transcatheter aspiration of vegetation) with inpatient mortality (defined as inpatient death or hospice discharge) and was adjusted for select demographic and clinical factors (Table 2). Only the 3 sites with available antibiotic data were included in the adjusted model.

**Results:**

169 cases of S-IE were identified with an overall increase from 2015 to 2021 and an apparent peak in 2019, although trends varied by site (Fig 1). 76% of cases were due to IDU, 56% involved a single left-sided valve and 40% were polymicrobial (Table 1). Inpatient mortality was 22%. 35% of patients had a procedural intervention, and 44% were treated with combination antibiotic therapy. Univariate analyses (Table 2) revealed significant associations between mortality and site of care, single left-sided valve disease, procedural management and treatment with combined beta-lactam and fluoroquinolone (BL+FQ). In the adjusted analysis, including 117 cases from 3 sites, lower inpatient mortality was associated with procedural intervention (OR 0.17, 95% CI 0.04-0.77) and BL+FQ treatment (OR 0.06, 95% CI 0.00-0.88).

**Figure:**
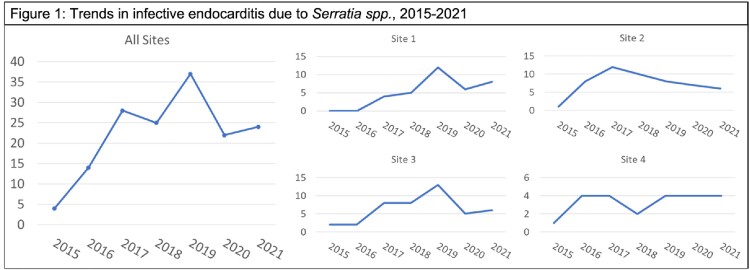

**Figure:**
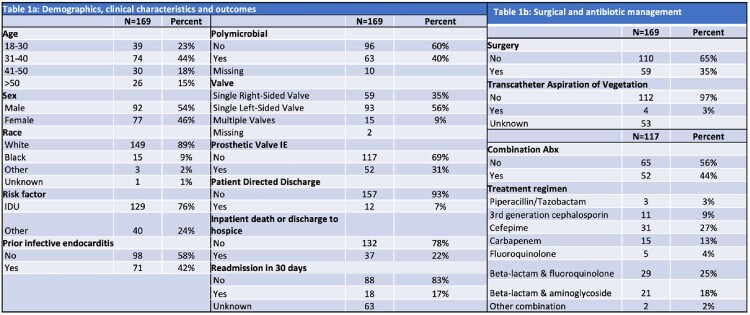

**Figure:**
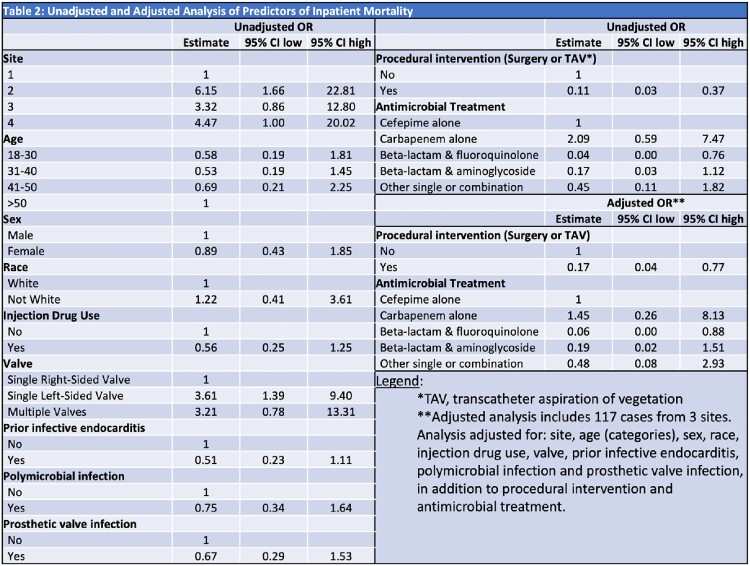

**Conclusion:**

In this large multicenter study, we found that S-IE is not infrequent, has high inpatient mortality and is treated with varying strategies. Procedural intervention and combination BL+FQ treatment were associated with lower mortality. Our study is limited by varying methods of case identification and a lack of data on clinical severity and surgical indications. Further study is urgently needed to define best management practices.

**Disclosures:**

**Asher J. Schranz, MD, MPH**, WoltersKluwer: Honoraria

